# A novel tetraspanin-related gene signature for predicting prognosis and immune invasion status of lung adenocarcinoma

**DOI:** 10.1007/s00432-023-05176-1

**Published:** 2023-07-30

**Authors:** Yindong Zhu, Ying Yang, Yuan Liu, Hongyan Qian, Ganlin Qu, Weidong Shi, Jun Liu

**Affiliations:** 1 Department of Oncology, Affiliated Hospital of Nantong University, Medical School of Nantong University, Nantong, China; 2 Cancer Research Center Nantong, Nantong Tumor Hospital, The Affiliated Tumor Hospital of Nantong University, Nantong University, Nantong, China; 3 Department of Thoracic Surgery, The Second People Hospital of Nantong, Nantong, China

**Keywords:** Lung adenocarcinoma, Tetraspanin-related genes, Tumor microenvironment, Prognosis, Gene signature

## Abstract

**Background:**

Lung adenocarcinoma (LUAD), the most common subtype of lung cancer, is the primary contributor to cancer-linked fatalities. Dysregulation in the proliferation of cells and death is primarily involved in its development. Recently, tetraspanins, a group of transmembrane proteins, have gained increasing attention for their potential role in the progression of LUAD. Hence, our endeavor involved the development of a novel tetraspanin-based model for the prognostication of lung cancer.

**Methods:**

A comprehensive set of bioinformatics tools was utilized to evaluate the expression of tetraspanin-related genes and assess their significance regarding prognosis. Hence, a robust risk signature was established through machine learning. The prognosis-predictive value of the signature was evaluated in terms of clinical application, functional enrichment, and the immune landscape.

**Results:**

The research first identified differential expression of tetraspanin genes in patients with LUAD via publicly available databases. The resulting data were indicative of the value that nine of them held regarding prognosis. Five distinct elements were utilized in the establishment of a tetraspanin-related model (*TSPAN7, TSPAN11, TSPAN14, UPK1B,* and *UPK1A*). Furthermore, as per the median risk scores, the participants were classified into high- and low-risk groups. The model was validated using inner and outer validation sets. Notably, consensus clustering and prognostic score grouping analysis revealed that tetraspanin-related features affect tumor prognosis by modulating tumor immunity. A nomogram based on the tetraspanin gene was constructed with the aim of enhancing the poor prognosis of high-risk groups and facilitating clinical application.

**Conclusion:**

Through machine learning algorithms and in vitro experiments, a novel tetraspanin-associated signature was developed and validated for survival prediction in patients with LUAD that reflects tumor immune infiltration. This could potentially provide new and improved measures for diagnosis and therapeutic interventions for LUAD.

## Introduction

Globally, lung cancer exhibits the highest mortality rate and ranks second in terms of its incidence rate. The 5-year survival rate for this disease is only 26% (Ganti et al. 2021). Among non-small cell lung cancer (NSCLC) subtypes, lung adenocarcinoma (LUAD) emerges as the most common, contributing to more than 40% of all lung cancer cases (Kim et al. 2020). Despite considerable advancements in therapeutic measures, such as surgical interventions, radiotherapy, and chemotherapy, individuals with LUAD still exhibit unfavorable prognoses. In recent years, the advent of immune checkpoint inhibitor (ICI) therapy has revolutionized the treatment of cancer by targeting immune checkpoints (Lin et al. 2019). Nonetheless, it must be noted that merely a small proportion of diseased individuals have benefited from ICI treatment.

To improve the prognosis and treatment of LUAD, identifying more potential indicators that can serve as novel therapeutic targets is crucial in this period of individualized therapy. Classical clinical models premised on tumor extension, TNM staging, performance status, and pathological staging indicators, for predicting the prognosis of LUAD individuals. However, the heterogeneity of LUAD hinders their performance, limiting their ability to attain satisfactory results (Zuo et al. 2019). Hence, the development of new models becomes imperative for enhancing the therapeutic measures and prognostic performance of LUAD.

The protein family, tetraspanins, is distinct due to the presence of four transmembrane segments, a large extracellular loop, and a small extracellular structural domain (Charin et al. 2014; Florin and Lang 1140). The small intracellular domains comprise palmitoylated cysteines and N- and C-terminal tails. Moreover, the homology between isoforms is highly conserved, with the exception of a small variable domain present within the large extracellular loop (Seigneuret et al. 2001). Tetraspanin proteins are highly conserved across species, with 33 of the 34 four-transmembrane proteins found in mammals also being present in humans (Beckwith et al. 2015). A notable link has been noted between the transmembrane tetraspanin proteins and various processes, including those involved in cancer, immunity, fertility, and infectious diseases. In oncogenesis, the gene family tetraspanin is known to impact the growth of tumors by influencing processes such as immune function, platelet coagulation, angiogenesis, and infection (Hemler 2008).

The current prevailing theory suggests that the prognosis of patients with LUAD is strongly linked to their immune infiltration and microenvironment. Although several studies have established the link of tetraspanins to the immune function of cancer, their ability to influence the prognosis of individuals with LUAD is yet to be elucidated (Hemler 2008). Hence, exploring the prognostic role of tetraspanin-related genes in LUAD may be critical.

Herein, five tetraspanin-related genes (TPRGs) associated with LUAD prognosis were identified. These findings may facilitate the development of therapeutic and diagnostic measures for individuals with LUAD.

## Methods

### Collection and processing of LUAD datasets

The respective websites, TCGA (https://portal.gdc.cancer.gov/) and the University of California Santa Cruz Xena, were searched to obtain the RNA sequencing (HTSeq-Counts) and copy number variation (CNV) information of LUAD. Individuals who lacked survival data were excluded from further analyses. To ensure that the samples were comparable, the HTSeq-Count data underwent normalization to transcripts per kilobase million (TPM) values. Furthermore, log2TPM transformation of the data was executed for subsequent analysis (Wagner et al. 2012).

The GEO database (https://www.ncbi.nlm.nih.gov/geo/) was searched to obtain the microarray data. Specifically, the datasets GSE30219 (*n* = 83) and GSE50081 (*n* = 127) from the GPL570 platform were utilized. They contained the matrix files of the complete clinical information and gene expression data. The transcriptomic data of the GEO cohort underwent log2-transformation. To address any possible batch effects arising from non-biological technical biases the “SVA” “ComBat” algorithm was utilized.

### Gene set variation analysis

The LUAD metabolic heterogeneity was explored through enrichment analysis by means of gene set variation analysis (GSVA) across various groups or patterns using the R “GSVA”. The resulting data were illustrated in the heatmap (Hänzelmann et al. 2013). The database MSigDB was searched to acquire the files “c2.cp. Kegg.v2022.1.Hs.symbols” and “c5.go.v2022.1.Hs.symbols” for GSVA. As per the adjusted *P*-value < 0.05, statistically significant pathways between diverse clusters were obtained.

### Estimation of infiltrating cells in tumor microenvironment

The various groups or clusters were assessed for significant variations in the tumor microenvironment (TME) infiltration of cells through a single-sample gene set enrichment analysis (ssGSEA). The process involved assessing the 28 subpopulations of tumor-infiltrating lymphocytes and quantifying their relative abundance in the LUAD TME for further differential analysis. Notably, the study encompassed various key subtypes of immune cells in humans including activated dendritic cells, activated CD4 + T cells, natural killer T cells, activated CD8 + T cells, and macrophages (Barbie et al. 2009; Charoentong et al. 2017).

### Association of molecular patterns with the clinical characteristics and prognosis of LUAD

The clinical significance of clusters established through consensus clustering was assessed by exploring the link between molecular patterns, clinical traits, and survival results. Age, gender, N- and T-stages, were included in the clinical factors. Additionally, Kaplan–Meier (KM) analysis was utilized to examine the variations in overall survival (OS) between distinct patterns through “survival” and “survminer” (Rich et al. 2010).

### Association of molecular patterns and TME in LUAD

ESTIMATE was employed to examine the immune and stromal scores of individuals with LUAD (Meng et al. 2020). Additionally, CIBERSORT was utilized to examine the levels of 22 subtypes of immune cells in every individual (Chen et al. 2018). Moreover, the proportion of infiltrated immune cells was determined through ssGSEA (Huang et al. 2021). The relationship between the two subsets was assessed in terms of the expression of PD-1, PD-L1, and CTLA-4.

### Development of the tetraspanin-associated prognostic TPRG_Score

The quantitative assessment of tetraspanins was executed using a TPRG_score in every individual with LUAD. The transcriptomic data of TPRGs from different clusters across LUAD specimens were standardized, followed by intersecting genes selection. Univariate Cox regression (uniCox) analysis was executed for TPRGs and the resulting survival-linked genes were examined further. Principal component analysis (PCA) was conducted for generating tetraspanin-linked gene scores utilizing the formula mentioned: TPRG_score = expression of a gene [1] × corresponding coefficient [1] + expression of a gene [2] × corresponding coefficient [2] + expression of the gene [n] × corresponding coefficient [n].

### Clinical significance and classification analysis based on TPRG prognostic signature

The relevance of the TPRG_score to clinical variables was examined. Furthermore, uniCox and multivariate Cox regression analyses were conducted for all cohorts to determine the capability of TPRG_score to independently predict the prognosis. Subsequently, the reliability and predictive capacities of the TPRG_score were examined in distinct subgroups (as per various clinical factors) through classification analysis. Additionally, the association of TPRG_score with cancer stem cell (CSC), microsatellite instability (MSI), and tumor mutation burden (TMB) scores was examined.

### Construction of a predictive nomogram

A nomogram was developed in order to improve the predictions provided for individuals with LUAD at the clinical level. This nomogram integrates risk scores and various other clinicopathological features, with a particular focus on predicting 1-, 3-, and 5-year OS. Subsequently, its clinical use and reliability were assessed through calibration curve analysis and decision curve analyses.

### Mutation and drug sensitivity analysis

The mutational information of individuals with LUAD across various risk groups was determined by utilizing the TCGA database to generate a mutation annotation format using “maftools” (Mayakonda et al. 2018). Moreover, the values for semi-inhibitory concentration (IC50) of commonly prescribed drugs were computed through “oncoPredict” to examine the clinical effectiveness of chemotherapeutic drugs in diseased individuals (Maeser et al. 2021).

### Statistical analysis

The processing of data, as well as their analysis and presentation, was executed through R v 4.2.3 and its relevant packages. A two-sided *P* < 0.05 was deemed as a statistically significant difference.

## Results

### Genetic mutational landscape of TPRGs in LUAD

**Fig. 1:**
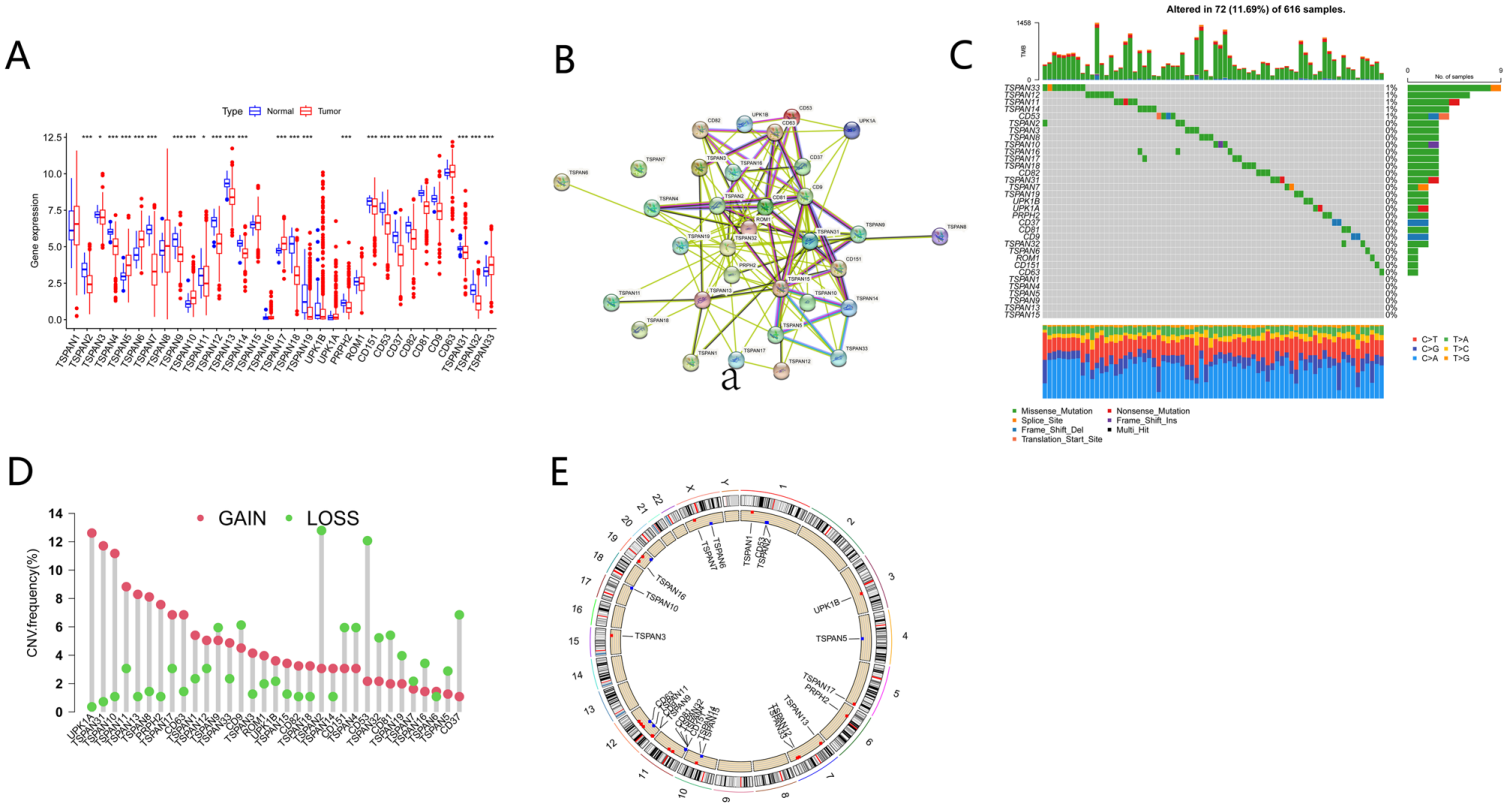
Genetic mutational landscape of TPRGs in LUAD. (**A**) DEG expression distribution between normal and GC tissues. (**B**) Protein–protein interaction network acquired from the STRING database among the TPRGs. (**C**) Genetic alteration of a query of TPRGs. (**D**) Non-CNV and CNV gain and loss frequencies among TPRGs. (**E**) TPRGs chromosome distribution illustrated through circus plots. *P* < 0.05 *; *P* < 0.01 **; *P* < 0.001 ***

**Fig. 2:**
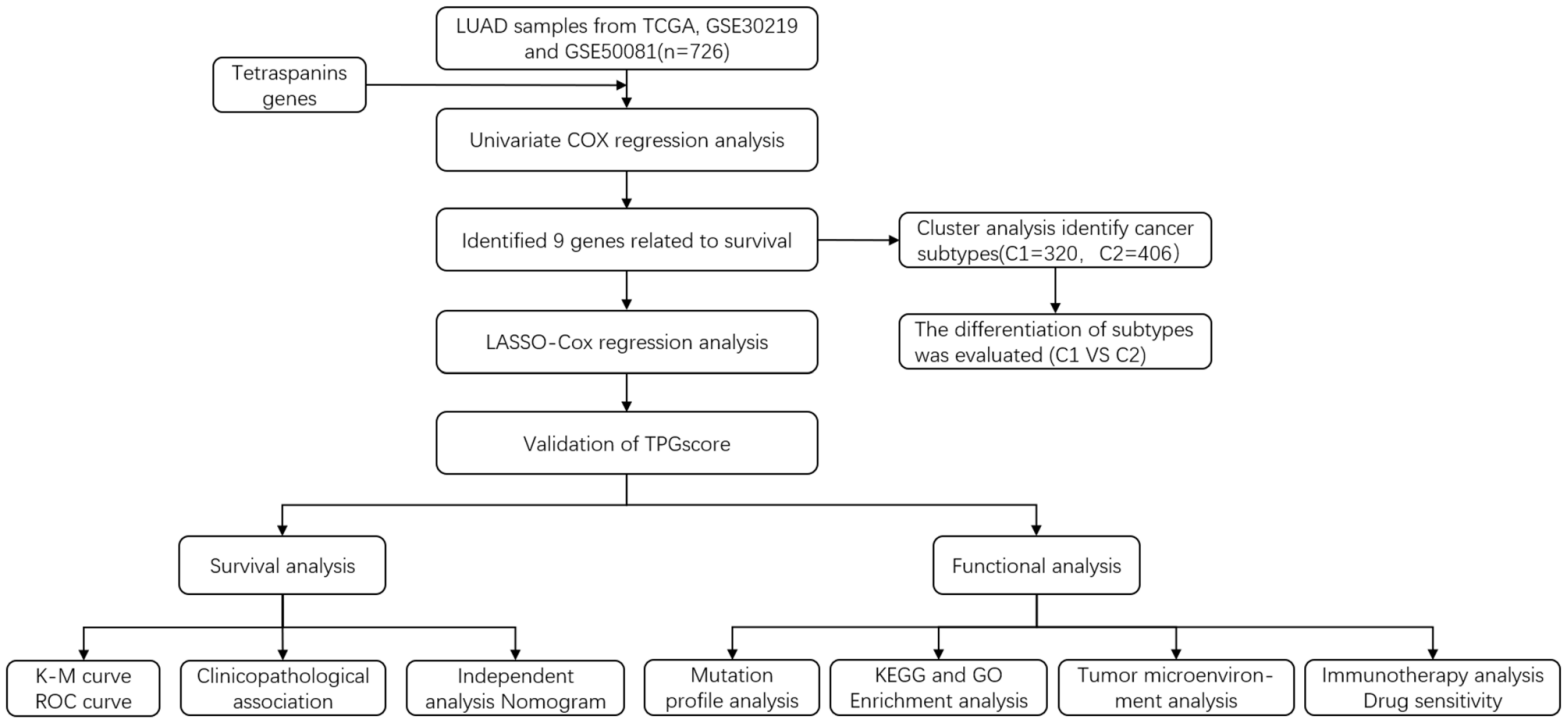
Study flowchart

The initial assessment of the levels of the 33 TPRGs concerning their expression in both tumor and normal specimens was carried out using the TCGA-LUAD dataset. Overall 26 differentially expressed genes (DEGs) were discovered, with the majority prevalent in the tumor samples (Fig. 1A). To examine the interactivity of these DEGs, a protein–protein interaction analysis was executed by employing the string website, which indicated that *TSPAN9*, *TSPAN14*, *TSPAN31*, *TSPAN32*, *TSPAN33*, and *CD9* were hub genes (Fig. 1B). Subsequently, on determining the occurrence of CNVs and somatic mutations of the 33 TPRGs in LUAD, it was found that 72 of the 616 (11.69%) LUAD samples presented genetic mutations. *TSPAN32* had the highest mutation incidence, followed by *TSPAN12* and *TSPAN11* (Fig. 1C). Furthermore, evident CNV alterations were noted in the 33 TPRGs (Fig. 1D), and the site of these alterations was mapped to specific chromosomes (Fig. 2E). The resulting data suggest that CNV may regulate the expression of TPRGs. Moreover, the data indicate substantial differences in the genomic background and levels of TPRG expression between LUAD and normal specimens, implying the possible involvement of TPRGs in LUAD tumorigenesis.

### Generation of tetraspanin subgroups in LUAD

**Fig. 3:**
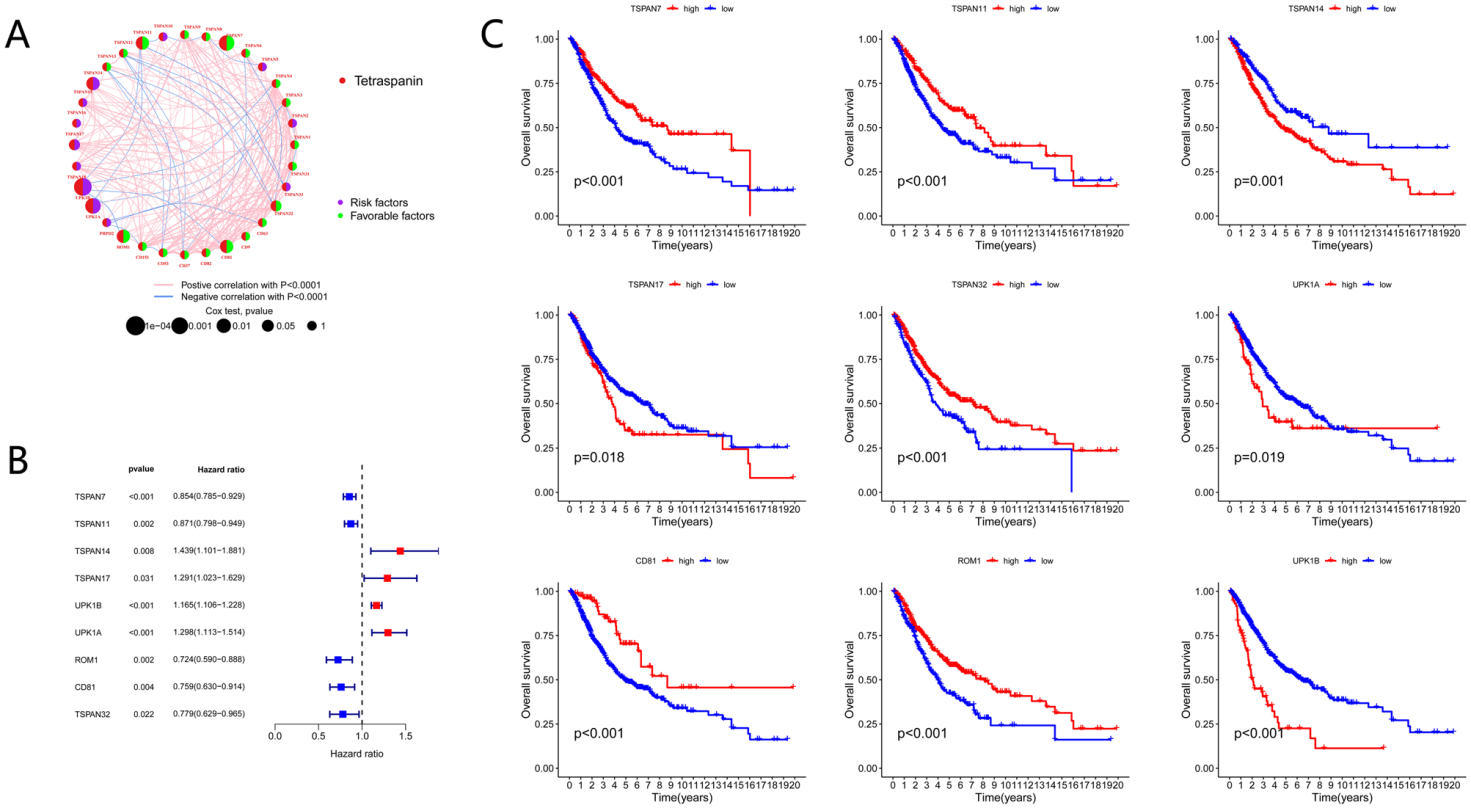
Survival analysis of TPRGs in LUAD. (**A**) Correlation network including TPRGs in TCGA cohort. (**B**) Univariate analysis showing nine TPRGs correlated with OS. (**C**) Kaplan–Meier analysis showing nine TPRGs correlated with OS

Figure 2 presents the research flowchart in detail. Herein, the data of 726 individuals with LUAD from TCGA–LUAD, GSE50081, and GSE30219 datasets were included to investigate the relationship between tetraspanins and tumorigenesis. The prognosis-predictive values of 33 TPRGSs were determined in individuals with LUAD using uniCox and Kaplan–Meier analyses (Fig. 3C). Additionally, a correlation network was generated encompassing TPRG interactions, regulator association, and their significance in terms of survival among individuals with LUAD (Fig. 3A).

**Fig. 4:**
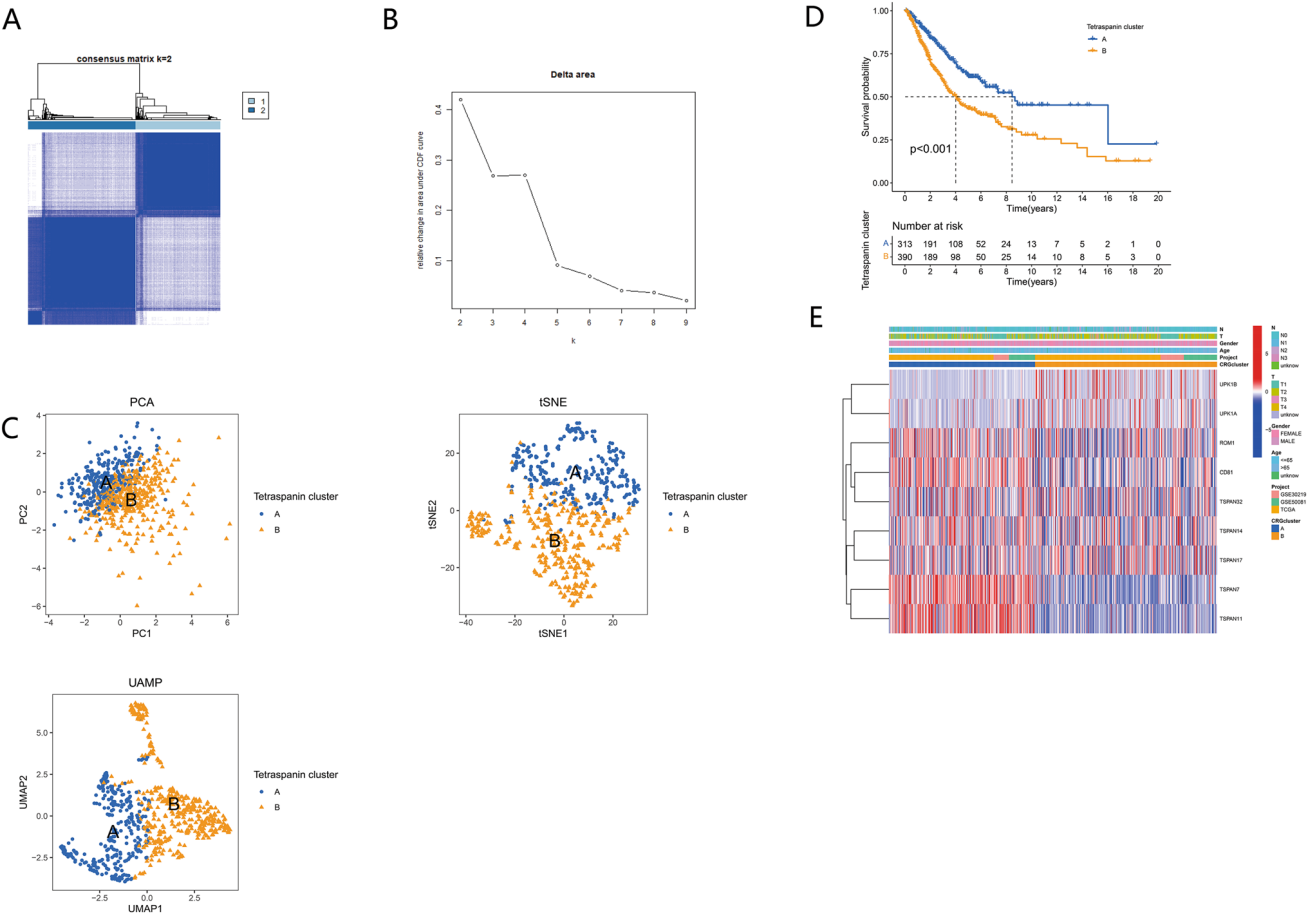
TPRG subtypes and clinicopathological and biological features of two distinct subtypes of samples divided by consistent clustering. (**A**) Two clusters (*K* = 2) and their correlation area defined through a heatmap of the consensus matrix. (**B**) Relative change area under cumulative distribution function curve. (**C**) PCA, tSNE, and UAMP depicted remarkable variations in transcriptome across the subtypes. (**D**) Kaplan–Meier curve indicated remarkable survival variations between clusters A and B (*P* < 0.001). (**E**) Variation in clinicopathologic parameters and levels of TPRG expression across the subtypes

To gain a further understanding of the functional biological pattern of these TPRGs as well as their significance at the clinical level, consistent clustering was executed. As per the expression levels of 16 TPRGs, the samples of the TCGA–LUAD cohort were classified into subgroups. The clustering stability was considered to be best when *K* = 2, as it provided stable clustering results from *k* = 2 to *k* = 9. Furthermore, the categorization of the TCGA–LUAD cohort was executed as per two distinct TPRGs (Fig. 4A, B) into TPRG clusters A (*n* = 320) and B (*n* = 406). Moreover, per the level of TPRG expression, the two groups depicted remarkable variations in the transcriptional profiles of TPRGs using PCA (Fig. 4C). Further survival analysis depicted an improved OS for the individuals in cluster A of TPRG than the other cluster (Fig. 4D). Moreover, both clusters were comparatively assessed concerning clinicopathological parameters and genomic expression (Fig. 4E). The resulting data depicted a remarkable variation in the two above-mentioned elements.

### Enrichment analysis based on consensus cluster

**Fig. 5:**
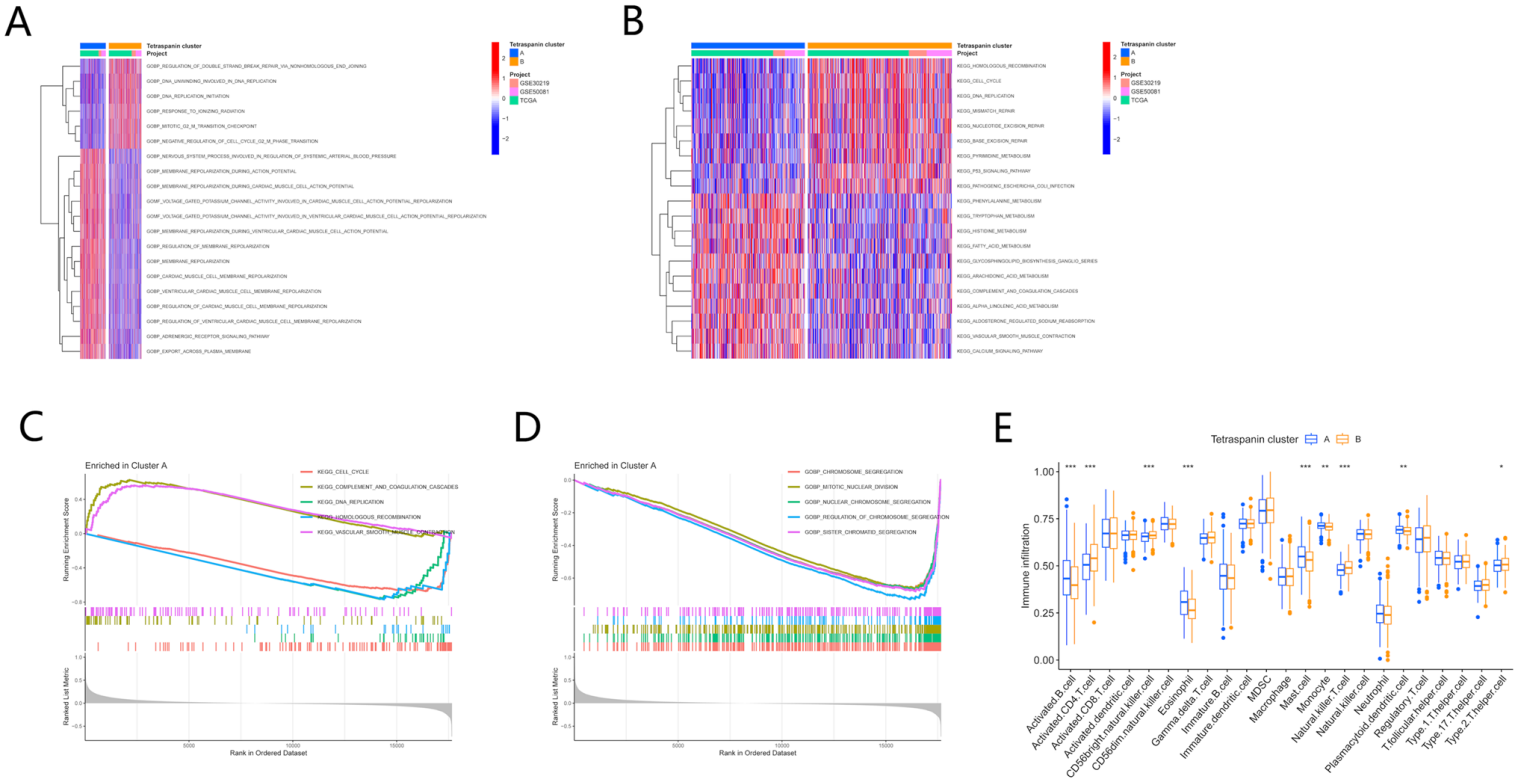
Enrichment function analysis based on consistent clustering. (**A**) GSVA of biological pathways across the two subtypes. The respective activated and inhibited pathways indicated by red and blue. (**B**) GSVA of gene function between two subtypes. Respective activated and inhibited pathways denoted by red and blue. (**C**) GSEA of biological pathways in cluster A. (**D**) GSEA of gene function in cluster A. (**E**) Infiltrating immune cell (23 types) abundance in the two subgroups

As per the resulting data of GSVA, cluster A was more prevalent in metabolism-linked pathways, including phenylalanine, tryptophan, histidine, fatty acid, and alpha-linolenic acid metabolism pathways (Fig. 5B). In contrast, cluster B was abundant in genetic information processing pathways, for instance, cell cycle, nucleotide excision repair, DNA replication, base excision repair, and mismatch repair. The GSVA functional enrichment analysis indicated the enrichment of cluster A primarily in biological processes and molecular functions linked to myocardial cell membrane repolarization, whereas cluster B depicted enrichment primarily in processes linked to cell division and regulation of DNA replication (Fig. 5A and Table S5). The results of the GSEA functional analysis revealed that chromosome segregation, mitotic nuclear fission, nuclear chromosome segregation, regulation of chromosome segregation, and sister chromatid segregation were primarily enriched in cluster B (Fig. 5C). In terms of pathway enrichment, complement and coagulation cascades and vascular smooth muscle contraction were enriched in cluster A, whereas DNA replication, cell cycle, and homologous recombination depicted enrichment in cluster B (Fig. 5D). A substantial difference in enrichment levels of the majority of immune cells between the two clusters was observed (Fig. 5E). Particularly, the enrichment levels of activated B cells, mast cells, eosinophils, monocytes, and plasmacytoid dendritic cells were remarkably elevated in cluster A in comparison to cluster B, whereas the activated CD4 + T, CD56^bright^ natural killer, natural killer T, and Type 2 T helper cells depicted the opposite trend.

### Development and validation of the prognostic TPRG_Score

**Fig. 6:**
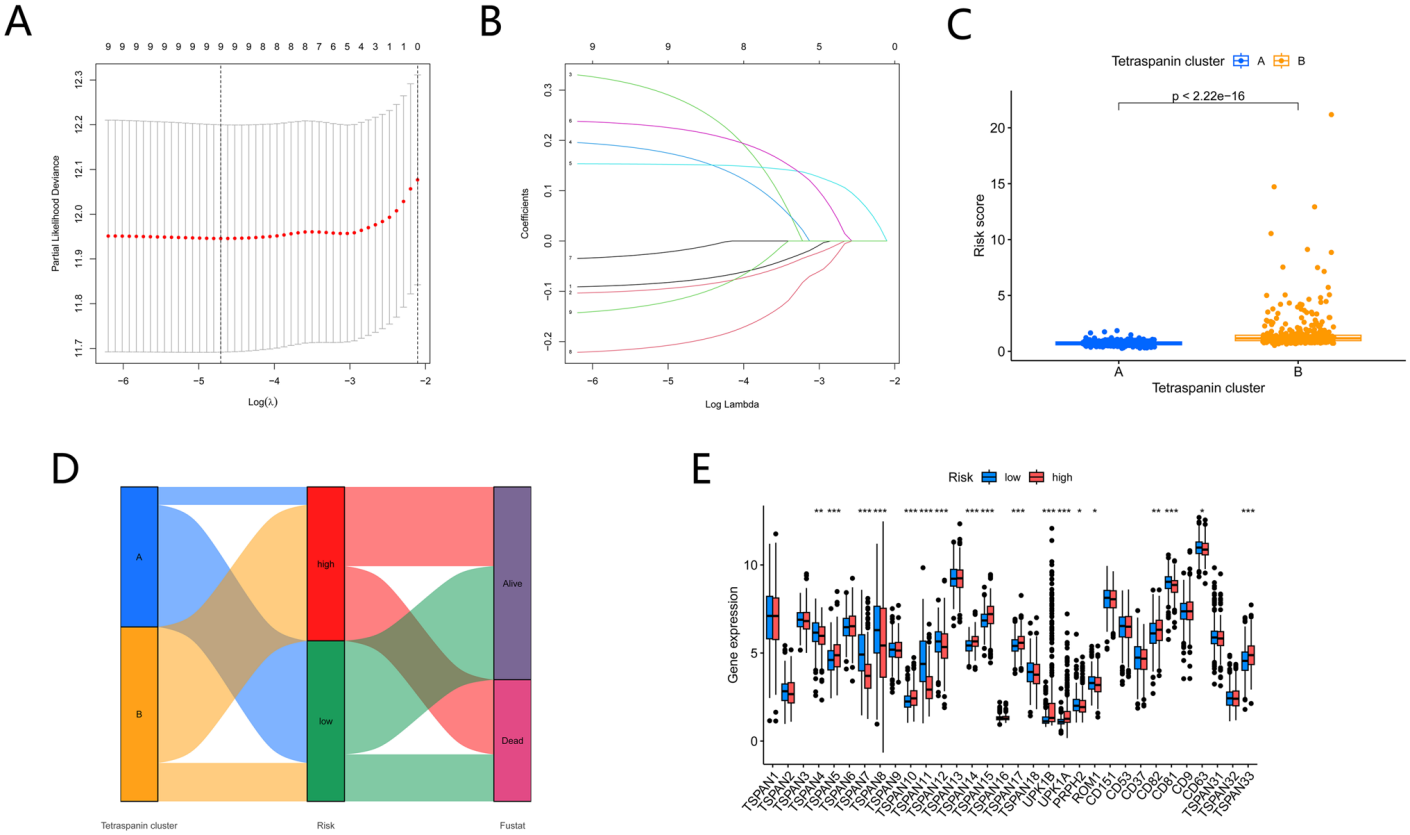
Establishment of the prognosis risk prediction model. (**A**, **B**) The least absolute shrinkage and selection operator regression was conducted with the minimum criteria. (**C**) Variation in TPRG_score across the two subtypes (**E**) Variation in clinicopathologic variables and level of TPRG expression across the risk groups (high- and low-risk). (**F**) Alluvial diagram of subtype distributions in groups with diverse TPRG_scores and survival outcomes

The TPRG_score was generated based on prognosis-associated TPRGs. The randomly assigned individuals with LUAD formed the training (*n* = 352) or test (*n* = 351) cohorts at a ratio of 1:1. An optimal predictive model was established by executing LASSO and multivariate Cox analysis for nine prognosis-linked TPRGs (Fig. 6A, B). Five genes (*TSPAN7*, *TSPAN11*, *TSPAN14*, *UPK1B*, and *UPK1A*) were acquired and the TPRG_score was quantified as mentioned below: Risk score = (− 0.1320*expression of *TSPAN7*) + (− 0.1281*expression of *TSPAN11*) + (0.3451*expression of *TSPAN14*) + (0.1740*expression of *UPK1B*) + (0.2498*expression of UPK1A). A remarkable variation in the TPRG_score of the tetraspanin clusters was noted (Fig. 6C). Figure 6D illustrates the distribution of the patients in the two tetraspanin clusters and two TPRG_score groups. Significant expression of genes related to the TPRG_score was observed in both TPRG gene clusters and was consistent with the expected results for a subset of TPRG_score (Fig. 6E).

### Prognostic significance assessment of the risk model in the training, test, and entire cohorts

**Fig. 7:**
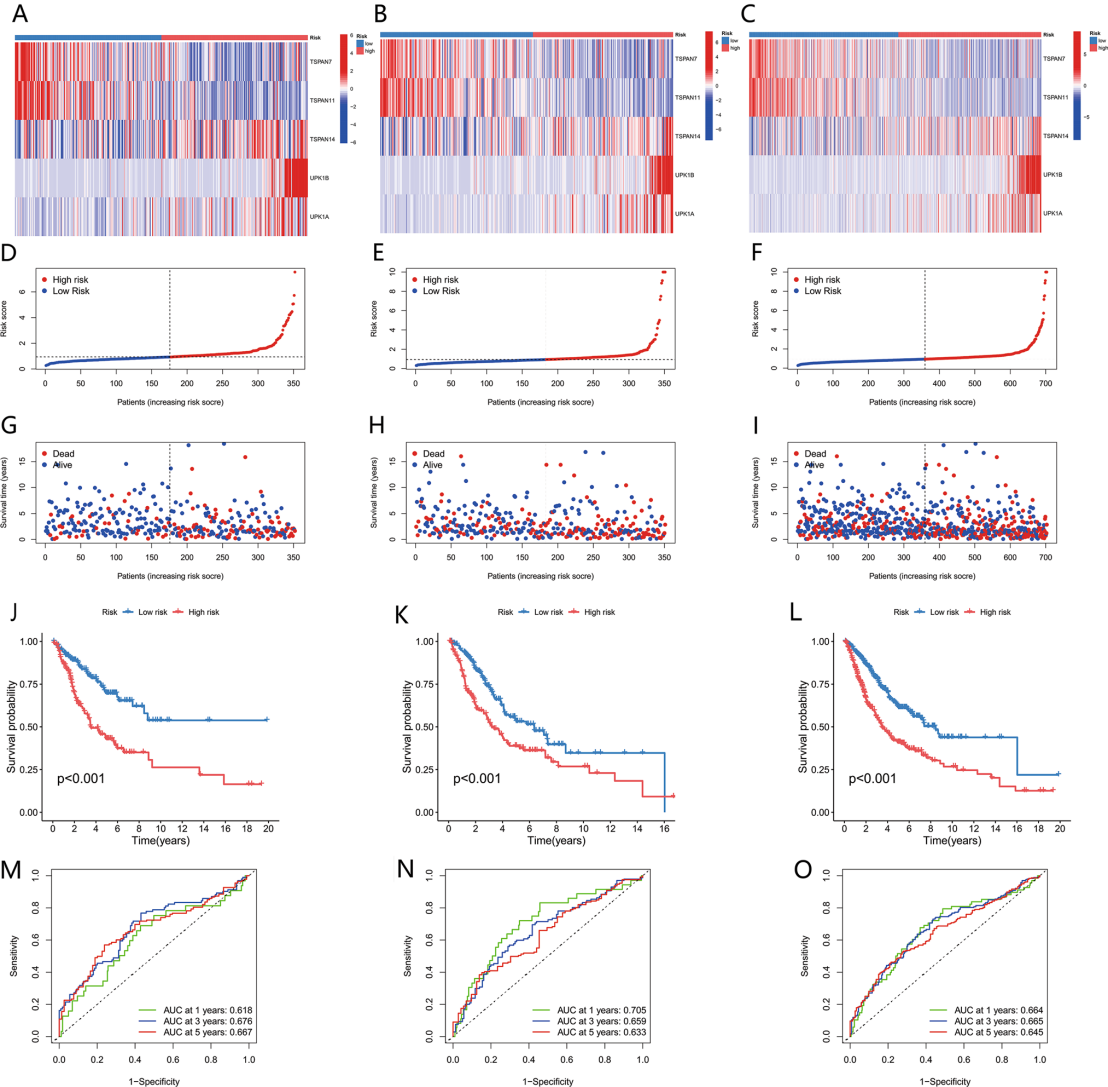
Assessment of the train, test, and entire sets for the prognostic value of the risk model. (**A**–**C**) Patterns of expression of five selected prognostic genes in the high- and low-risk groups. (**D**–**F**) Display of the TPRG model as per the risk score of the train, test, and entire sets, respectively. (**G**–**I**) Comparison of survival time and status across the risk groups (low and high) in the three sets, respectively. (**J**–**L**) Kaplan–Meier survival curves showing the survival probability of patients between the risk groups in the three sets, respectively. (**M**–**O**) ROC curves for specificity and sensitivity prediction of 1-, 3-, and 5-year survival as per the TPRG_score in the three sets, respectively

The three cohorts, training set A (*n* = 352), verification set B (*n* = 351), and entire set C (*n* = 703) were comparatively assessed regarding the distribution of risk score, survival probability, survival status, and expression level of related genes between the risk subgroups (low and high). The resulting data indicated an unfavorable prognosis for the subgroups with high risk (Fig. 7A–L). The model was assessed regarding its performance by plotting a time-dependent ROC curve with AUC being computed at various time points. The respective AUC values for sets A, B, and C were 0.618, 0.705, and 0.664 at 1 year; 0.676, 0.659, and 0.665 at 3 years; and 0.667, 0.633, and 0.645 at 5 years (Fig. 7M–O). The resulting data indicated the strong prognosis-predictive value of the model concerning follow-up encompassing both short- and long-term periods.

### Construction of a nomogram for prognostic prediction in patients

**Fig. 8:**
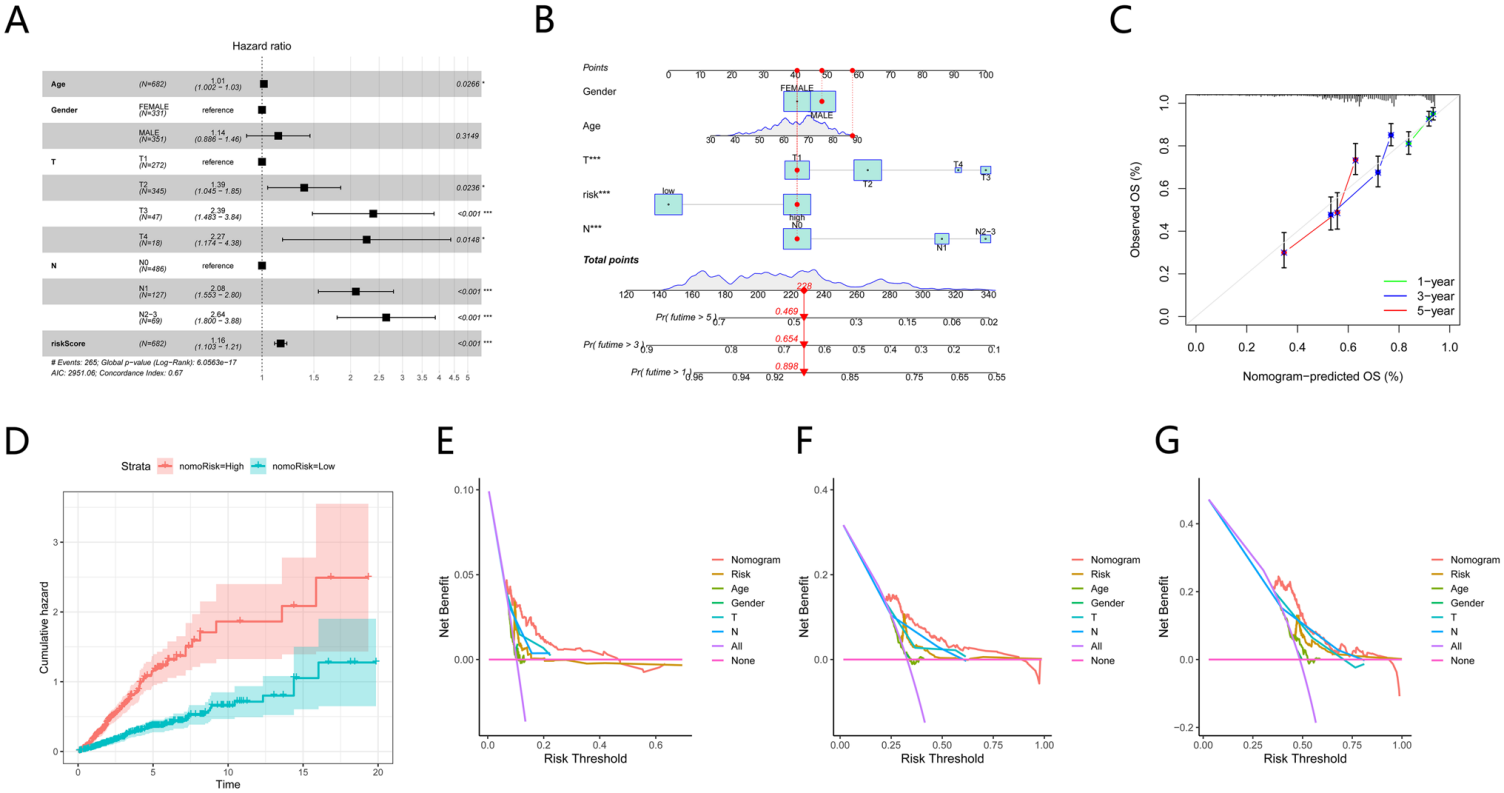
Development and validation of a nomogram. (**A**) Multivariate Cox regression analyses. (**B**) 1-, 3-, and 5-year OS prediction of individuals with GC in the entire cohort through nomogram. (**C**) ROC curves for 1-, 3-, and 5-year ROC prediction in the entire cohort. (**D**) Cumulative hazard of the two nomogram cohorts. (**E**) Decision curve analysis curves of the nomograms comparatively assessed for 1-, 3-, and 5-year OS in HCC

Taking into account patient age and tumor stage, the Cox regression analyses (univariate and multivariate) determined that tumor stage and TPRG_score have independent predictive capabilities concerning OS in individuals with LUAD (Fig. 8A). In order to develop a nomogram, clinical variables were incorporated, given the strong association of risk scores with patient prognosis. The 1-, 3-, and 5-year OS values were estimated for individuals with LUAD through the nomogram (Fig. 8B). A close alignment between the predicted values and the actual observations was depicted through calibration curves of this established nomogram (Fig. 8C). Over time, the risk increased, and the individuals from the high-risk group were at high risk in comparison to those from the group with low risk (Fig. 8D). Moreover, measurements were taken for these clinical factors regarding their respective AUC values at 1, 3, and 5 years for predicting OS. These values were consistent with expectations, indicative of the remarkable predictive capacity of the nomogram for prognosis. Moreover, this prognostic model incorporating diverse clinical factors presented more net benefits for predicting the patient prognosis (Fig. 8E–G).

### Correlation of TPRG_score with TMB, CSC score, and checkpoints in distinct groups

**Fig. 9:**
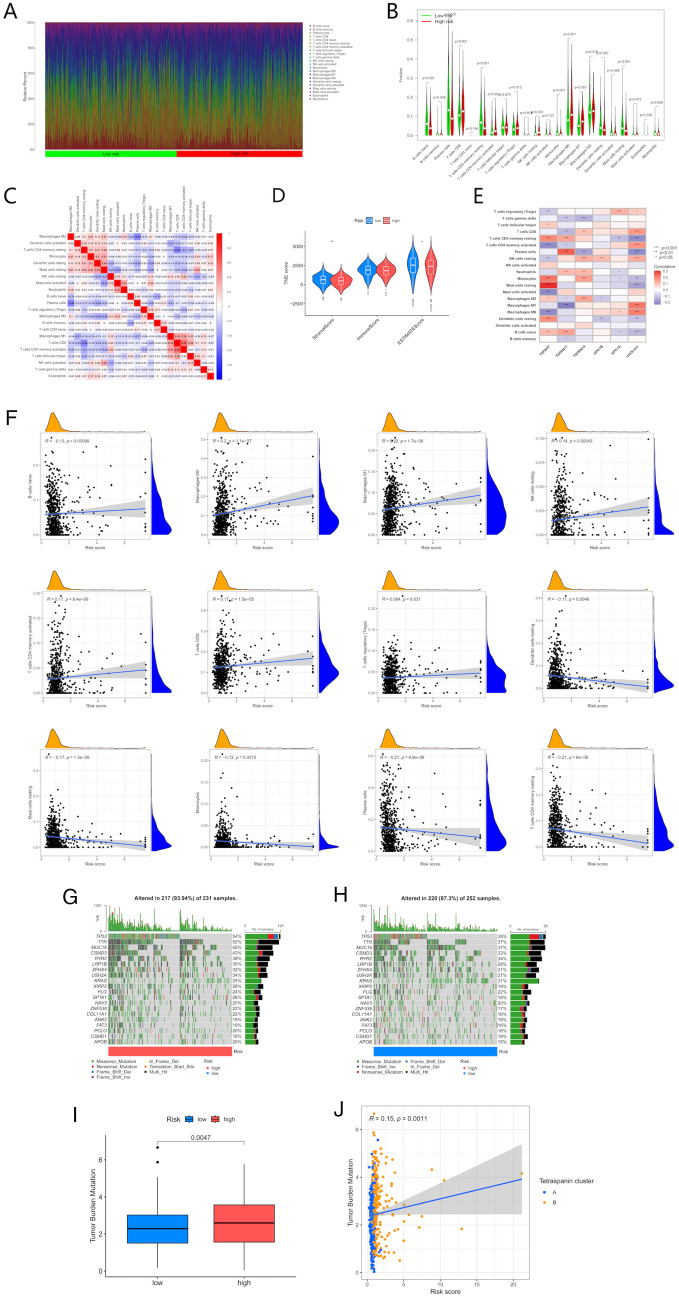
Evaluation of TMB, CSC score, and checkpoints between the two groups. (**A**, **B**) Immune infiltration analysis showing the percentage abundance of tumor-infiltrating immune cells between risk scores (high and low) in individuals with LUAD. (**C**) Correlation analysis of 22 tumor-infiltrating immune cell risk scores. (**D**) Correlation analysis between TPRG_score and stromal and immune scores. (**E**) Associations between immune cell abundance and selected genes in the prognostic model. (**F**) Correlation analysis between TPRG_score and immune cell types. (**G**, **H**) Waterfall plot showing somatic mutation characteristics in TPRG_score (high and low) groups. (**I**, **J**) Correlation analysis between TPRG_score and TMB

The immune microenvironment substantially influences both the development of lung adenocarcinoma and the effectiveness of immunotherapy. To study this relationship, the TME of individuals with LUAD was examined. The risk groups (high and low) were assessed through CIBERSORT to examine the relative percentage of invasive immune cells. A ranking of low to high risk scores for the LUAD samples depicted the percentage of various immune cells (Fig. 9A). TPRG_score was positively linked to the M1 macrophages, M0 macrophages, activated memory CD4 + T cells, CD8 + T cells, resting natural killer cells, and regulatory T cell infiltration. In contrast, the opposite trend was noted in association with TPRG_score and resting dendritic cells, naive B cells, monocytes, plasma cells, resting mast cells, and resting memory CD4 + T cells (Fig. 9F). The association of the selected genes in the predictive signature with immune cell enrichment was examined. The resulting data indicated that TSPAN7, TSPAN11, and TSPAN14 were strongly linked to the genes that were selected (Fig. 9E). Furthermore, the TPRG_score was negatively associated with immune and stromal scores (Fig. 9D). Most immune cells varied between the risk groups (high- and low) concerning their infiltration profiles (Fig. 9B). Research has depicted the remarkable prognosis-predictive functions of TMB and MSI as indicators for tumor immune response. ICP inhibitors can prove favorable in individuals with elevated TMB or MSI (31–33). These outcomes exhibited a positive correlation between TMB and risk level, depicting increased levels in the individuals with high risk than the ones at low risk (Fig. 9H). The data are indicative of the more favorable influence that immunotherapeutic measures may have on high-risk individuals. A positive association between TPRG_score and TMB was noted through Spearman correlation analysis (Fig. 9I). Moreover, utilizing the TCGA–STAD dataset, the variation in the distribution of the somatic mutations between TPRG_score patterns was examined. *TP53, TTN, MUC16, CSMD3, RYR2,* and *ZFHX4* in the two risk groups depicted a mutation incidence of equal to or higher than 30% in individuals with LUAD (Fig. 9F, G). Notably, a higher likelihood of mutations was noted in the high-risk group relative to the other group.

### Anticancer drug sensitivity assessment in individuals with varied TPRG_scores

**Fig. 10:**
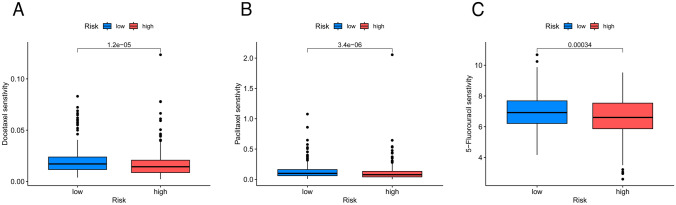
Comparison of susceptibility to chemotherapy drugs between high- and low-risk groups. (**A**–**C**) Comparison of susceptibility to docetaxel, paclitaxel, and 5-fluorouracil, respectively

The low- and high-risk populations were assessed concerning their susceptibility to the selected anti-cancer drugs. The high TPRG individuals depicted decreased IC50 values of docetaxel, paclitaxel, and 5-fluorouracil compared to those with lower scores. Collectively, these data suggest a potential association of the TPRGs with susceptibility to drugs (Fig. 10).

## Discussion

In this research, nine tetraspanin genes were identified in LUAD that were associated with prognosis through a prognostic analysis. The cohort was sorted into two clusters through the K-means algorithm, and the tetraspanin gene set in LUAD was determined to be primarily enriched in metabolism-linked and genetic information processing pathways. Cluster B had a significantly lower OS than Cluster A. Five TPRGs were obtained through univariate regression, LASSO, and stepwise regression analysis. They were then employed for establishing a new prognostic risk signature to sort patients with LUAD into high- and low-risk subgroups. The prognostic signature integrated five TPRGs, namely TSPAN7, TSPAN11, TSPAN14, UPK1B, and UPK1A. The data depicted that TSPAN7 and TSPAN11 were linked to a favorable prognosis in LUAD, whereas TSPAN14, UPK1B, and UPK1A were linked to an adverse prognosis. These findings could lead to improvements in the early diagnosis methods for lung cancer patients, as earlier diagnosis often leads to better treatment outcomes.

The resulting data validated that the adverse prognosis of individuals with lung malignancy was linked to elevated expression levels of TSPAN7. Previous reports have pointed out that direct correlations were noted between TSPAN7 upregulation and advanced tumor stage, lymph node status, and adenocarcinoma. Additionally, TSPAN7 promotes epithelial–mesenchymal transition and stimulates an increase in the N-cadherin expression level in NSCLC cells by attenuating the expression of E-cadherin and vimentin (Wang et al. 2018). The expression level of TSPAN14 was reduced in malignant cells than in non-tumor cells. Reduced gene expression was linked to unfavorable patient survival and was more common in individuals with aggressive tumor types. Reduced expression of TSPAN14 leads to enhanced expression of MMP-2 and MMP-9 (matrix-degrading enzymes), resulting in the enhanced ability of cancer cells to degrade the matrix (Jovanović et al. 2022). But TSPAN11, UPK1B, and UPK1A are currently not related to lung cancer research. In brief, these findings indicate that while our study identified five genes suitable for the development of prognostic markers, not all of them have been previously linked to lung cancer. This observation may be attributed not only to the structural similarity but also to the functional diversity of the tetraspanin family. These results highlight the significant involvement of these five TPRGs in LUAD and demonstrate their high sensitivity and specificity in identifying LUAD patients. This also could provide a clue for new lung cancer treatment strategies. For instance, if we could find a way to reduce the expression of TSPAN9 or restore the expression of TSPAN14, it might help to inhibit further progression of the tumor.

TPRG_score was significantly correlated with the clinicopathological traits of LUAD. Upon adjusting for confounding variables, the resulting data indicated the capacity of TPRG_score to independently predict survival outcomes among individuals with LUAD. Additionally, its predictive robustness was further validated upon executing ROC displaying the 1-, 3-, and 5-year OS. This means that the TPRG score can be utilized to help us predict a patient's prognosis more accurately, and adjust the treatment strategies based on these predictions. For example, those with a high TPRG score may need a more aggressive treatment strategy, while those with a low TPRG score may be suitable for a more conservative treatment approach.

As LUADs are becoming increasingly resistant to chemotherapy (Rossi and Maio 2016), promising sensitive drugs were determined in diverse TPRG_score patient groups in the present study. Paclitaxel and docetaxel are presently considered the primary drugs for first-line lung cancer chemotherapy, while 5-fluorouracil has not been endorsed by current treatment guidelines for lung cancer. The data depicted that targeting tetraspanins in combination with these drugs offers potential benefits in mitigating drug resistance and improving clinical outcomes, providing a new strategy for lung cancer treatment and new drug development targets for pharmaceutical companies.

Furthermore, specific biomarkers as predictive models are required to ensure the effectiveness of immunotherapy. The accumulation of genetic alteration leads to carcinogenesis, which is linked to immune infiltration. The resulting data are indicative of the remarkable variation in genomic alterations between TPRG scores (low and high). Increased TMB is correlated with a better prognosis of immunotherapy in individuals with LUAD, hence depicting congruence with the data of this research (Negrao et al. 2021). The clinical outcome in the low TPRG_score group was remarkably more favorable in contrast with that in the low TMB group, indicating that TPRG_score can be employed for independently predicting the response to immunotherapeutic interventions. A notable finding from prior research indicated a negative association of tumor purity with immune response. The data exhibited that tumor purity could function as a proxy for the degree of immune response in the microenvironment of the tumor (Liu et al. 2019). Congruent with these data, the clinical outcome in the low TPRG_score group was remarkably more favorable with that in the low TMB group, elevated levels of different immune cell infiltrations were exhibited in the low-TPRG score group, indicating that TPRG_score could be utilized to identify individuals who better respond to tumor immunotherapy. This discovery proposes that, in a clinical setting, we can utilize the TPRG_score as a predictive indicator of immunotherapy effectiveness, thereby informing our decision-making process regarding the administration of immunotherapeutic drugs to patients.

There are several noteworthy limitations to consider. First, the hypothesis requires additional validation through further research. Second, the cohorts were limited to TCGA, GEO, and Nantong, which restricts the ability to fully evaluate the quality of the data. To more comprehensively assess the prognosis-predictive cuproptosis-signature, a multicenter prospective, study is necessary. Lastly, conducting in vivo experiments may provide additional clarity regarding the processes and functional role of the cuproptosis-signature in the onset and progression of LUAD.

In summary, the research dealt with developing a prognostic tetraspanin-associated signature that could be utilized for predicting survival among individuals with LUAD by characterizing tumor immune infiltration. The data indicated that immunotherapy is crucial to improving the prognosis of patients with LUAD. Thus, the research findings are anticipated to offer valuable insights into precisely diagnosing and treating patients with LUAD.

## Data Availability

Data are available in a public, open access repository. Data are available upon reasonable request. All data relevant to the study are included in the article.
